# What would primary care practitioners do differently after a delayed cancer diagnosis? Learning lessons from their experiences

**DOI:** 10.1080/02813432.2023.2296117

**Published:** 2024-02-07

**Authors:** Tuomas H. Koskela, Magdalena Esteva, Marcello Mangione, Sara Contreras Martos, Senada Hajdarevic, Cecilia Högberg, Mercè Marzo-Castillejo, Jolanta Sawicka-Powierza, Vija Siliņa, Michael Harris, Davorina Petek

**Affiliations:** a Faculty of Medicine and Health Technology, Tampere University, Tampere, Finland; b Center of General Practice, Tampere University Hospital, Tampere, Finland; c Health Research Institute of the Balearic Islands (IdISBa), Palma, Spain; d Local Health Authority Committee, Palermo City, Italy; e Research Support Unit Metropolitana Sud, University Institute for Primary Health Care Research IDIAPJordi Gol, Catalan Health Institute, Barcelona, Spain; f Department of Nursing, Umeå University, Umeå, Sweden; g Department of Public Health and Clinical Medicine, Family Medicine, Umeå University, Umeå, Sweden; h Department of Public Health and Clinical Medicine, Unit of Research, Education and Development Östersund, Umeå University, Umeå, Sweden; i Department of Family Medicine, Medical University of Białystok, Białystok, Poland; j Department of Family Medicine, Riga Stradiņš University, Riga, Latvia; k Institute of Primary Health Care Bern (BIHAM), University of Bern, Bern, Switzerland; l College of Medicine & Health, University of Exeter, Exeter, UK; m Department of Family Medicine, Faculty of Medicine, University of Ljubljana, Ljubljana, Slovenia

**Keywords:** Cancer, diagnostic errors, Europe, primary care physicians, primary health care, qualitative research

## Abstract

**Objective:**

Diagnosis of cancer is challenging in primary care due to the low incidence of cancer cases in primary care practice. A prolonged diagnostic interval may be due to doctor, patient or system factors, or may be due to the characteristics of the cancer itself. The objective of this study was to learn from Primary Care Physicians’ (PCP) experiences of incidents when they had failed to think of, or act on, a cancer diagnosis.

**Design:**

A qualitative, online survey eliciting PCP narratives. Thematic analysis was used to analyse the data.

**Setting and subjects:**

A primary care study, with narratives from 159 PCPs in 23 European countries.

**Main outcome measures:**

PCPs’ narratives on the question ‘If you saw this patient with cancer presenting in the same way today, what would you do differently?

**Results:**

The main themes identified were: thinking broadly; improvement in communication and clinical management; use of other available resources and ‘I wouldn’t do anything differently’.

**Conclusion (Implications):**

To achieve more timely cancer diagnosis, PCPs need to provide a long-term, holistic and active approach with effective communication, and to ensure shared decision-making, follow-up and continuing re-assessment of the patients’ clinical conditions.

## KEY POINTS

- Diagnosing cancer in primary care is challenging due to the low incidence of cancer in practice and the multiple confounding factors that are involved in the diagnostic process.
- The need to think broadly, make improvements in communication and clinical management, and use other available resources were the main themes from Primary Care Physicians’ (PCPs’) narratives about their learning experiences from missed or late cancer diagnoses.
- A long-term, holistic and active approach with effective communication, follow-up and continuing re-assessment of the patients’ clinical conditions was another theme for making improvements.
- Some PCPs, on reflection, would not have done anything differently.

## Introduction

The diagnosis of cancer in symptomatic patients requires a complex multi-step process, involving multiple factors from patients, doctors and health care systems. Being at a point of first medical contact, as well as having a unique longitudinal doctor-patient relationship over time, Primary Care Physicians (PCPs) are well placed to make prompt cancer diagnoses and detect cancer early. However, there are particular difficulties in diagnosing cancer in primary care, where, for individual doctors, a diagnosis of cancer is a rare event [1]. PCPs typically diagnose only one case of each of the most common cancers (colorectal, prostate, breast, and lung) each year, but less frequent cancers might be seen only once or twice during a PCP’s career [1]. Cancer is not a single disease but a heterogeneous entity which comprises many different types of disease, and the type and intensity of signs and symptoms can vary greatly from patient to patient and evolve over time [2]. Many signs and symptoms, even the ones considered as ‘red flags’ of alarm, have low positive predictive values for cancer diagnosis [3]. Vague symptoms [4] and multimorbidity [5] can contribute to lower suspicions of cancer and thereby prolonged time to diagnosis. In addition, many PCPs do not have direct access to some of the diagnostic technologies and must refer their patients to specialists, which can cause delays in investigations and consultations. All these factors can result in misdiagnoses or avoidable delays in diagnosis [4, 5], increasing the probability of advanced stage and poorer prognosis [6].

In a British study, PCPs assessed that one in four cancer cases had an avoidable delay in diagnosis [5]. There can be missed opportunities in all the different phases of the diagnostic process: during the initial assessment, diagnostic test performance and interpretation, and coordination and follow-up [4, 5, 7].

Missed diagnostic opportunities are considered to have happened when something different could have been done to make the correct diagnosis earlier [8]. One of the gaps in research recognized by the World Health Organizationin in its report on diagnostic errors is in evidence on the most practical and effective methods of providing feedback to providers, and how to implement systems that encourage providers and systems to learn from diagnostic errors [9]. In recent years, qualitative research has identified some of the diagnostic pitfalls in primary care diagnosis of cancer. These studies have provided insights into the presentations of the patients diagnosed with cancer as a result of an emergency admission [10], in cognitive errors in clinical reasoning [11], the impacts of communication with patients [12], and in symptom appraisal for a specific type of cancer [13]. PCPs’ focus on feedback and error analysis has been suggested as a tool to improve performance, with the potential to transform errors into learning opportunities [8].

PCPs’ reflection on possible missed opportunities concerning cancer diagnosis should help to identify multiple factors, not only those associated with their own performance, but also those linked with health system and patient factors that may impede them from making a timely cancer diagnosis. This study therefore examines PCPs’ views on what they would do differently after they had failed to think of, or act on, a cancer diagnosis.

## Material and methods

### Study design

In this qualitative study we used an online survey with open-ended questions asking PCPs for their narratives about a delayed cancer diagnosis. We used thematic analysis as it enabled us to identify patterns of meaning across a set of data and explore PCPs experiences, actions and reflections on the clinical cases that they presented, and allowed us to take into account the contexts in which the events were placed.

### Setting

A multicentre online survey of PCPs in 23 European countries in 2021, led by 11 research group members.

### Development of the questionnaire

The Örenäs Research Group (ÖRG) is a European collaborative of 94 primary care researchers in 32 countries, formed in 2013 to study the factors influencing national variations in the early diagnosis of cancer in primary care. In 2018, a core study group of ÖRG members agreed on the research question ‘Why do European PCPs sometimes not think of, or act on, a possible cancer diagnosis?’ The group wrote the text for the survey questions, which included demographic questions, and piloted it to test the questions, assess whether the survey instructions were clear and understandable, and estimate how many survey responses would be needed to achieve data saturation. Fourteen other ÖRG members completed the pilot questionnaire, giving 15 narratives in total. Thematic analysis of the pilot survey responses resulted in 43 codes and 4 themes; saturation was not achieved in the pilot. No question misunderstandings were detected.

The questionnaire invited participants to submit a narrative: ‘Please write a short description of a time when you were slow to think of a cancer diagnosis, or where you thought of cancer but were slow to do something about it.’, followed by the free-text questions: ‘What happened?’, and ‘Why do you think it happened?’. As a result of the pilot, the core group added a new question about what PCPs would do differently as a result of their experiences, and in this paper we focus on their answers to this question:

If you saw this patient presenting in the same way today, what would you do differently?

The questionnaire also asked for PCPs’ demographic data: country, gender, whether they were a trainee, years of working experience (≤4 years, 5–14 years, ≥15 years) and practice setting (town/city, rural, island/remote, or mixed). To increase anonymity, the answer ‘I prefer not to say’ was also given as an option for all the questions except for the country. PCPs were also asked to name the type of cancer in the case that they were describing. The questionnaire was put online using SurveyMonkey (SurveyMonkey, California, USA).

### Participants

Study subjects were General Practitioners (GPs) and doctors who had other specialist training but worked in the community and could be accessed directly by patients without referral, here collectively referred to as Primary Care Practitioners (PCPs). We aimed for least 5 PCP responses, with a maximum of 10, from each of the 23 participating countries. To achieve maximum variation, the study purposefully included a balance of female and male PCPs, a range of years of experience, and different practice locations (both rural and non-rural). However, these targets were flexible, as they depended on the availability of respondents in each country. The pilot study suggested that this sample size would achieve data saturation.

### Recruitment and data collection

In some countries the ÖRG local leads chose to invite possible participants themselves; in the other countries, the local leads identified PCPs that might be interested in the study and sent their contact details to the research group leader, MH, who invited them to join the study.

Invitation emails, in participants’ native languages, were sent to potential PCP respondents. The invitation email described the objectives and methodology of the study and explained that all data would be collected anonymously: the identity of participating physicians would not be identifiable, and IP addresses would not be collected. A ‘Participant Information Sheet’ was included in the invitation email.

As there was a possibility that the meaning of the questions could change when translated into other languages, the survey questions were in English for all participants. Leads were allowed to translate the text into their local languages in their invitation emails if they wished, but none chose to do so. Participating PCPs were asked to answer the questions either in their own languages or, if they felt confident to do so, in English. In order to preserve patient anonymity, participants were asked to describe their cases in such a way that neither their own identities nor those of their patients could be inferred from their descriptions. MH downloaded the survey results and confirmed that the data preserved anonymity. Consent was implied by agreeing to take part in the survey. Each survey response was given an identification code. Answers not in English were sent to the local lead who translated them into English, with the help of English native or professional translators. Respondents’ demographic data was removed before being sent to translators.

### Analysis of data

We used thematic analysis according to Braun and Clarke’s six-step framework [14], with open and cross coding, and hierarchical grouping into themes.

The research team consisted of 11 members, most of whom were experienced PCPs. In the first part of analysis, we divided the research team into three working groups. Each of these analysed 53, randomly assigned participants’ responses. Each researcher independently coded the text assigned to their group at two levels. Initially, each researcher read the text as a phase of familiarisation with the data. We then conducted initial coding by going through transcripts line by line, assigning codes to the text based on our interpretation of the text focusing on its underlying meaning. All the codes and the related citations were identified in Excel documents. The differences in researchers’ lower and preliminary higher-level codes were resolved in subgroup online meetings. The results of each subgroup’s preliminary coding were discussed at an online meeting of the entire ÖRG core study group, allowing development of a joint data-driven coding framework.

The next analytic step was to further group the codes into higher-level hierarchical units. The research team was then reorganised into two new subgroups, one of which, with five members, focused on the answers to the third free-text question: ‘… what would you do differently?’, which is reported here. The thematic analysis of the other questions, ‘What happened?’ and ‘Why do you think it happened?’, has been reported elsewhere [15].

The group focusing on the question ‘…what would you do differently?’ organised data into sub-themes and themes. Each hierarchical level was discussed within the subgroup at online meetings, and finally at two online meetings of the whole ÖRG core study group. Some discussions also took place by email. We sorted codes that we interpreted as belonging together into potential themes and sub-themes. Further, we created themes by clustering sub-themes that were related to each other. No software was used for data analysis due to the complexities of online cooperation across multinational researchers.

Finally, we looked back at the raw data to see how it supported the themes and our overarching theoretical perspective. We looked for original data citations that supported the analytical themes. We checked that every quote and lower code could be assigned to at least one theme, and that at least one quote could be found for every theme and sub-theme. The themes were discussed at an online meeting of the entire ÖRG core study group, and this resulted in small adjustments. We used the COREQ checklist as our reporting guideline [16].

## Results

In total,159 PCPs answered the questionnaire. One answer that did not include a case description was excluded. The analysis included 158 cancer cases from 23 European countries. Working experience after qualifying as a doctor was fifteen years and over for almost two thirds of the PCPs. Under a quarter of the responders were GP trainees. One third of the PCPs worked in rural or mixed areas. (Table 1)

**Table 1.** Characteristics of the primary care physicians that participated in the survey, N (%).

Total participants		158 (100)
Gender	Women	89 (56.3)
	Men	68 (43.0)
	Prefer not to say	1 (0.6)
Work experience	<4 years	15 (9.5)
	4–15 years	46 (29.1)
	>15 years	97 (61.4)
Training status	Established PCP	121 (76.6)
	GP trainee	37 (23.4)
Area of work	Town or city	99 (62.7)
	Rural	33 (20.9)
	Island or remote	5 (3.2)
	Mixed	20 (12.6)
	Prefer not to say	1 (0.6)
Country	Bulgaria	10 (6.3)
	Croatia	8 (5.1)
	England	7 (4.4)
	Estonia	6 (3.8)
	Finland	5 (3.2)
	Germany	5 (3.2)
	Greece	8 (5.1)
	Ireland	10 (6.3)
	Israel	4 (2.5)
	Italy	10 (6.3)
	Latvia	10 (6.3)
	Lithuania	6 (3.8)
	Netherlands	3 (1.9)
	Norway	7 (4.4)
	Poland	7 (4.4)
	Romania	8 (5.1)
	Scotland	5 (3.2)
	Slovenia	6 (3.8)
	Spain	14 (8.9)
	Sweden	8 (5.1)
	Switzerland	3 (1.9)
	Turkey	6 (3.8)
	Ukraine	2 (1.3)

Most (80%) of the respondents wrote their answers in English and the rest in their native languages.

The analysis resulted in five themes with several subthemes (Table 2). Many individual cases contained codes belonging to several subthemes and themes. The themes and subthemes are described below, with each PCP’s quotation identified by an anonymised code due to the sensitivity of our data.

**Table 2.** Themes and sub-themes.

Theme	Subtheme
Think broadly	‘Think cancer’ – always have cancer as a possible differential diagnosis. Respect your ‘gut feelings’. Think about the patient’s risk factors. Don’t rely on previous negative results or specialist opinions.
Improve communication with the patient	Listen to the patient carefully. Share your concerns, actions and potential outcomes with the patient. Be assertive with a patient if you suspect cancer. Be persistent with your message to the patient and provide detailed information
Improve clinical management	Check information about the patient carefully. Re-assess the patient if he/she doesn’t improve or if symptoms change. Do your clinical examination more carefully. Follow the patient actively. Practice continuity of care. If you suspect cancer, don’t watch and wait.
Use other available resources	Follow the clinical guidelines. Improve supportive diagnostic tools.
I would not do anything differently	

### Think broadly

This first theme covers PCP’s thoughts on the need to keep their thinking and diagnostic options broad enough. They reported that they were less likely to do this in younger patients, when the presentation was complex due to comorbidity, with rare cancers, or when patients were frequent attenders. They thought that it was important always to keep the possibility of cancer as a differential diagnostic possibility, and not to rely too much on others’ opinions:

It was really rare; cancer of pancreas in male <30y. (2D18)

I would probably order imaging tests earlier to exclude other causes than post-infection cough. (1D32)

Consider other diagnoses in the person who presents with the same symptom but where the hospital says there is nothing wrong. (3D17)

PCPs felt that they should take into account the potential risk factors for cancer and listen to their gut feelings:

Now if I see a patient who has had cancer, even if it was some years ago, I think about whether today’s symptoms could be because of a recurrence of cancer - and investigate if needed. (3D9)

Do not rely on the results from additional diagnostic investigations only, but focus more on [the doctor’s] own observations and his/her gut feeling. (2E53)

### Improve communication with the patient

The second theme focuses on the need to improve communication between PCPs and their patients. PCPs wrote about the basic elements of good communication, such as attentive listening to the patients’ symptoms and giving good explanations:

I would be more careful. I would pay more attention. Medical history is very important in these patients. (3D23)

The importance of negotiation, trust and the resulting agreement with the patient were emphasized:

Surely that experience taught me to emphasize suspicious clinical presentation and not to underestimate the power of the doctor-patient mutual trust. (3D21)

However, in addition, PCPs intended to be more assertive with their patients, and to be more persistent with their messages about necessary procedures:

I would stress what concerns me. (2E32)

Give more attention and time to explain and convince the patient to continue with evaluation. I’ll be more active. (3D19)

One PCP stated that they would be more willing and able to communicate clearly:

I have become more confident now. I think I would find the right words to persuade the patient. (1D46)

### Improve clinical management

This theme brings out, from the perspective of clinical management, several aspects which may hinder a PCP’s cancer diagnostic process, and, consequently those that could facilitate it if improved. PCPs’ suggestions were aimed at both individual and practice levels.

PCPs emphasised the need for sufficient time to evaluate the medical history, and to safety-net by monitoring and following-up their patients when needed:

Taking time to make a proper clinical history and differential diagnosis, making sure no serious conditions can be undiagnosed and, if something more serious is suspected, arrange a second appointment to address this case with more time. (2E41)

I would recall him (the patient) after a right period of 3 weeks of waiting, but I would be safer to act in a active way, that is to follow and call the patient if he will not come after the deadline. (3D22)

### Use other available resources

The fourth theme focuses on the potential for available resources. Guidelines were mentioned as a tool that helps PCPs to make a timely cancer diagnosis:

I think that the guidelines would now push me much more to refer with symptoms that I would have previously been happy to ‘watch and wait’. And of course having had this happen I probably started referring much more readily. (2E9)

The potential of electronic reminders to improve diagnostics was mentioned as well:

Improve informations in electronic health record like a summary of illness and follow-up visits/test plan - Activation of electronic health reminders for recommended testing. (2E38)

I would at least make automatic reminders to secure the control of the blood samples. (2E28)

### I would not do anything differently

Finally, despite everything, some PCPs felt that they wouldn’t do anything differently another time. These cases were mostly patients with rare cancers, such as sarcoma or renal cancer.

I am afraid I couldn’t have really done anything differently. (1D38)

## Discussion

### Statement of principal findings

In this qualitative study we found four themes describing what PCPs would do differently after having failed to think of, or act on, a possible cancer diagnosis: think broadly, improve communication and clinical management, and use other available resources. However, some participants would not change their actions. The themes are diverse and raise multiple aspects at both individual and practice levels.

### Findings in relation to other studies

#### Think broadly

The PCPs identified in our study the importance of thinking broadly, as diagnostic errors can occur when cancer is not considered in the differential diagnosis. Previous studies have found that PCPs’ diagnostic strategies can fail to identify cancer when patients present with atypical and rare presentations [17], younger patients, and patients with comorbidities [18]. Rare or frequent attenders are less likely to receive an urgent referral or be suspected of cancer [19]. In another study, PCPs recognised that there were complex situations in which the differential diagnosis could be clouded by coexisting morbidity, by other concurrent events, or by symptoms that initially improved with treatment [10].

Some PCPs in our study regretted not having acted on their sense of alarm, and doing so might have helped to trigger investigations for cancer [20]. This is supported by a Nordic study, in which some doctors recognised that intuitive feelings of alarm helped them to come to think of cancer during a clinical encounter [21].

#### Improve communication with the patient

In this theme, the PCPs explained that they would like to improve communication with patients in future to speed-up cancer diagnosis. The PCPs emphasised the importance of the basic elements of good communication: attentive listening, and adequate explanation and education, which have been mentioned in literature as being necessary for good patient adherence [22]. The quality of interaction between patient and doctor, and the doctor’s accuracy in perceiving and interpreting cues, could be decisive in raising awareness of cancer [21]. Good communication facilitates the description of symptoms and increases the accuracy of the diagnosis [23, 24], and this was also identified by our participants. Evidence suggests that the importance of communication, the negotiation and the resulting agreement between the patient and the doctor affect the patient’s decision to follow the proposed measures and to participate in the investigations [12]. Careful, patient-centred explanations and information-giving reduce patients’ anxiety, which could otherwise deter patients from participating in diagnostic follow-up [22]. Patients have stated that when their symptoms are not carefully addressed by their PCPs, their motivation for re-consulting is low [25]. PCPs in our study were also aware that effective communication takes time, and this has also been identified in other studies [23, 24].

PCPs’ emphasis on assertiveness was a particularly interesting finding in our analysis. Assertive communication is essential for patient safety and refers to specific observations and making decisions from positional authority of the physician [26]. Our PCPs expressed their intention to be more assertive and persistent in the future, and they attributed the delay in cancer diagnosis to a lack of convincing communication with the patient. In this sense, assertiveness in communication is an important tool for effective transfer of doctor’s knowledge and expertise to the patients [23] in order to achieve their compliance.

#### Improve clinical management

Our respondents emphasised the importance of active follow-up and continuity of care. There is strong evidence for the benefits of continuity of care on clinical outcomes, service use and mortality in primary care [27], although its impact on the cancer diagnostic process may be small [28]. This theme includes safety-netting, which has become an integral part of clinical care in a variety of settings, for example specific information for patients on how and when to seek help, a plan for re-assessment of patient’s condition, and follow-up of investigations and hospital letters [7, 29].

The need for careful familiarisation with the patient’s previous medical history, as well as a clinical examination of the patient, were identified by some respondents. Recognising warning symptoms and signs observed in clinical examination could be decisive in the PCP’s clinical reasoning in relation to cancer [30]. Fast action using rapid pathways when cancer is suspected were also mentioned by our PCPs, and this maps across to previous ÖRG research [24]. These points are concordant with the Nordic core values and principles for family medicine, especially with values 1 (*We promote continuity of doctor-patients care as a central organising principle*) and 2 (*We provide timely diagnosis and avoid unnecessary tests and overtreatment…*) [31].

#### Use other available resources

Some of our PCPs mentioned that clinical guidelines could be helpful to trigger a referral when alarm symptoms appear, instead of ‘wait and see’. Guidelines have been introduced in several European countries to help with the process of symptom evaluation and earlier diagnosis of cancer [32, 33]. However, sometimes doctors can be reluctant to follow guidelines, as shown by research where six out of ten patients presenting to primary care with high-risk symptoms of a possible cancer did not receive an urgent referral [17]. This could be due to PCPs not always being convinced of the benefits of guidelines’ recommendations, or because they feel under pressure to have a low referral rate and to avoid over-diagnosis [7].

Our PCPs’ wish for supportive tools that could be helpful for recognising alarm symptoms and for patient management map across to a study showing that clinical decision support systems have potential to increase adherence to guidelines [34]. However, use of artificial intelligence in cancer diagnostics in primary care is at an early stage, and there is a lack of evidence on its performance in primary care setting, barriers to its implementation, and concerns about its cost-effectiveness [35].

#### I wouldn’t do anything differently

Some PCPs stated that they would not do anything differently next time, stating that they had made the right decision. This could be due to low-risk symptoms, rare cancers, and patients’ reluctance to seek medical attention [36]. It has been suggested that the presence of nonspecific symptoms, the absence of other risk factors and being in low-risk groups understandably do not prompt immediate diagnosis, and that ‘watchful waiting’ is one of the recognised strategies of work in primary care [7].

### Strengths and weaknesses of the study

This is the first multinational study focusing on experiences of PCPs who self-identified lessons learned from a case they failed to think of, or act on, a possible cancer diagnosis. It offers a comprehensive insight into the lessons to be learned from cases of participants from 23 European countries, covering different health care systems, work experiences and practice settings. Our multinational research group developed and piloted the questions before use and worked together for the coding and the thematic analysis. Using this method we were able to be aware of the cultural and health care peculiarities of the participating countries. The large range of participating countries means that the identified themes are likely to be relevant to PCPs in a variety of countries and healthcare systems.

Ideas for improvement were focused on the clinical cases and may not have reflected participants’ broader views. The PCPs were asked what they themselves would do differently, so their views are their reflections on their own actions rather than their thoughts on the need for any system changes. This means that their proposals represent only one aspect of improvements needed for more timely cancer detection. Their choice of cases was subjective and could have been their most memorable or recent cases, not those with the strongest implications for their future professional behaviour. Many of their narratives were short, which may have hampered the ability to put them into context.

Some participants answered in English, which was not their native language, and some answered in their own language, in which case their answers were translated by their national lead. This could have resulted in linguistic inaccuracies, although when recruiting participants we sought PCPs who were likely to understand the English-language questions. Any linguistic misunderstandings between members of our multilingual research group could have had an impact on our interpretation of the data. We gathered rich data from 158 PCPs and all their responses were analysed. Even though there were no in-depth interviews, similar comments were repeated many times by respondents from different countries. This suggests that it is unlikely that new themes would have emerged with additional responders, indicating data saturation.

### Meaning of the study: possible mechanisms and implications for clinicians or policy makers

Our findings have implications for PCP training and postgraduate education. PCPs should be encouraged not to rely on the simplest and most obvious explanation for a patient’s symptoms, but also to think broadly about several differential diagnostic possibilities. PCPs need to feel diagnostic insecurity if the patients’ symptoms do not improve with negative tests, and this should remain even if the specialist’s opinion is that cancer is unlikely. Gut feelings with a sense of alarm can contribute to the timely diagnostic process and should not be neglected. Continuity of care and an active follow-up of patients are especially important in unclear diagnostic situations with common and generic symptoms. Guidelines and digital solutions may facilitate timely diagnosis and reassessments: these should be developed and used. Active listening, including the patient in the conversation, as well as a careful and thorough explanation, are important for getting the patient’s perspective and a shared view with the patient on a diagnostic plan. Being more assertive with the patient, and more persistence in ensuring joint agreement on further actions, may be necessary steps for an effective diagnostic process. However, sometimes PCPs must accept that, even with hindsight, it may be that no changes could have been usefully made to improve the cancer diagnostic process.

## Conclusions

PCPs identified a number of learning points following a failure to think of, or act on, a possible cancer diagnosis. These include the need to think more broadly in their clinical reasoning, and have a long-term, holistic and active approach with effective communication ensuring shared-decision making, follow-up, and continuing re-assessment of the patient’s clinical condition.

## Data Availability

To avoid the risk of identification of individual participants or patients, the datasets generated and analysed during the current study are not publicly available. However, they are available (with any identifying information redacted) from the corresponding author on reasonable request.

## References

[1] Rubin G, Berendsen A, Crawford SMM, et al. The expanding role of primary care in cancer control. Lancet Oncol. 2015;16(12):1231–1272. doi:10.1016/S1470-2045(15)00205-3. 26431866

[2] Cancer Research UK. https://www.cancerresearchuk.org/about-cancer/cancer-symptoms., Accessed June 8, 2023.

[3] Shapley M, Mansell G, Jordan JL, et al. Positive predictive values of 5% in primary care for cancer: systematic review. Br J Gen Pract. 2010;60(578):e366–77. doi:10.3399/bjgp10X515412. 20849687 PMC2930247

[4] Jensen H, Nissen A, Vedsted P. Quality deviations in cancer diagnosis:prevalence and time to diagnosis in general practice. Br J Gen Pract. 2014;64(619):e92–e98. doi:10.3399/bjgp14X677149. 24567622 PMC3905405

[5] Swann R, Lyratzopoulos G, Rubin G, et al. The frequency, nature and impact of GP-assessed avoidable delays in a population-based cohort of cancer patients. Cancer Epidemiol. 2020;64:101617. doi:10.1016/j.canep.2019.101617. 31810885

[6] Neal RD, Tharmanathan P, France B, et al. Is increased time to diagnosis and treatment in symptomatic cancer associated with poorer outcomes? Systematic review. Br J Cancer. 2015;112(Suppl 1):S92–S107. doi:10.1038/bjc.2015.48. 25734382 PMC4385982

[7] Lyratzopoulos G, Vedsted P, Singh H. Understanding missed opportunities for more timely diagnosis of cancer in symptomatic patients after presentation. Br J Cancer. 2015;112 Suppl 1(Suppl 1):S84–S91. doi:10.1038/bjc.2015.47. 25734393 PMC4385981

[8] Singh H, Schiff GD, Graber ML, et al. The global burden of diagnostic errors in primary care. BMJ Qual Saf. 2017;26(6):484–494. doi:10.1136/bmjqs-2016-005401. PMC5502242 27530239

[9] World Health Organization. Diagnostic errors: technical series on safer primary care. Geneva: world Health Organization; 2016. Licence: CC BY-NC-SA 3.0 IGO. Available at https://www.who.int/publications/i/item/9789241511636

[10] Mitchell ED, Rubin G, Merriman L, et al. The role of primary care in cancer diagnosis via emergency presentation: qualitative synthesis of significant event reports. Br J Cancer. 2015;112 Suppl 1((Suppl 1))::S50–S6. doi:10.1038/bjc.2015.42. 25734395 PMC4385976

[11] Goyder CR, Jones CHD, Heneghan CJ, et al. Missed opportunities for diagnosis: lessons learned from diagnostic errors in primary care. Br J Gen Pract. 2015;65(641):e838-44–e844. doi:10.3399/bjgp15X687889. 26622037 PMC4655738

[12] Amelung D, Whitaker KL, Lennard D, et al. Influence of doctor-patient conversations on behaviours of patients presenting to primary care with new or persistent symptoms: a video observation study. BMJ Qual Saf. 2020;29(3):198–208. doi:10.1136/bmjqs-2019-009485. PMC7057803 31326946

[13] Mills K, Birt L, Emery JD, et al. Understanding symptom appraisal and help-seeking in people with symptoms suggestive of pancreatic cancer: a qualitative study. BMJ Open. 2017;7(9):e015682. doi:10.1136/bmjopen-2016-015682. PMC5588944 28871013

[14] Braun V, Clarke V. Using thematic analysis in psychology. Qualitative Research in Psychology. 2006;3(2):77–101. doi:10.1191/1478088706qp063oa.

[15] Hajdarevic S, Högberg C, Marzo-Castillejo M, et al. Why do european primary care physicians sometimes not think of, or act on, a possible cancer diagnosis? A qualitative study. BJGP Open. 2023;: BJGPO.2023.0029. Jun 28:BJGPO.2023.0029. Epub ahead of print. PMID: 37380218.doi:10.3399/BJGPO.2023.0029. 37380218 PMC11176697

[16] Tong A, Sainsbury P, Craig J. Consolidated criteria for reporting qualitative research (COREQ): a 32-item checklist for interviews and focus groups. Int J Qual Health Care. 2007; Dec19(6):349–357. doi:10.1093/intqhc/mzm042. 17872937

[17] Kostopoulou O, Delaney BC, Munro CW. Diagnostic difficulty and error in primary care–a systematic review. Fam Pract. 2008;25(6):400–413. doi:10.1093/fampra/cmn071. 18842618

[18] Wiering B, Lyratzopoulos G, Hamilton W, et al. Concordance with urgent referral guidelines in patients presenting with any of six ‘alarm’ features of possible cancer: a retrospective cohort study using linked primary care records. BMJ Qual Saf. 2022;31(8):579–589. doi:10.1136/bmjqs-2021-013425. PMC9304100 34607914

[19] Jensen H, Hoffmann C, Merrild C, et al. Association between GPs’ suspicion of cancer and patients’ usual consultation pattern in primary care: a cross-sectional study. Br J Gen Pract. 2019;69(679):e80–e87. doi:10.3399/bjgp19X700769. 30642908 PMC6355274

[20] Hardy V, Yue A, Archer S, et al. Role of primary care physician factors on diagnostic testing and referral decisions for symptoms of possible cancer: a systematic review. BMJ Open. 2022;12(1):e053732. doi:10.1136/bmjopen-2021-053732. PMC8788239 35074817

[21] Johansen M-L, Holtedahl KA, Rudebeck CE. How does the thought of cancer arise in a general practice consultation? Interviews with GPs. Scand J Prim Health Care. 2012;30(3):135–140. doi:10.3109/02813432.2012.688701. 22747066 PMC3443936

[22] van Dam L, Korfage IJ, Kuipers EJ, et al. What influences the decision to participate in colorectal cancer screening with faecal occult blood testing and sigmoidoscopy? Eur J Cancer. 2013;49(10):2321–2330. doi:10.1016/j.ejca.2013.03.007. 23571149

[23] Popa-Velea O, Purcărea VL. Issues of therapeutic communication relevant for improving quality of care. J Med Life. 2014;7 Spec No. 4(Spec Iss 4):39–45. 27057247 PMC4813615

[24] Harris M, Thulesius H, Neves AL, et al. How european primary care practitioners think the timeliness of cancer diagnosis can be improved: a thematic analysis. BMJ Open. 2019;9(9):e030169. doi:10.1136/bmjopen-2019-030169. PMC6773305 31551382

[25] Walter FM, Penfold C, Joannides A, et al. Missed opportunities for diagnosing brain tumours in primary care: a qualitative study of patient experiences. Br J Gen Pract. 2019;69(681):e224–e235. doi:10.3399/bjgp19X701861. 30858332 PMC6428480

[26] Omura M, Maguire J, Levett-Jones T, et al. The effectiveness of assertiveness communication training programs for healthcare professionals and students: a systematic review. Int J Nurs Stud. 2017;76:120–128. doi:10.1016/j.ijnurstu.2017.09.001. 28964979

[27] Sandvik H, Hetlevik Ø, Blinkenberg J, et al. Continuity in general practice as predictor of mortality, acute hospitalization, and use of out-of-hours care: a registry-based observational study in Norway. Br J Gen Pract. 2022;72(715):72e84–72e90. doi:10.3399/BJGP.2021.0340. 34607797 PMC8510690

[28] Ridd MJ, Ferreira DL, Montgomery AA, et al. Patient-doctor continuity and diagnosis of cancer: electronic medical records study in general practice. Br J Gen Pract. 2015;65(634):e305-11–e311. doi:10.3399/bjgp15X684829. 25918335 PMC4408510

[29] Melnikow J, Chan BK, Stewart GK. Do follow-up recommendations for abnormal papanicolaou smears influence patient adherence? Arch Fam Med. 1999;8(6):510–514. doi:10.1001/archfami.8.6.510. 10575390

[30] Scheel BI, Holtedahl K. Symptoms, signs, and tests: the general practitioner’s comprehensive approach towards a cancer diagnosis. Scand J Prim Health Care. 2015;33(3):170–177. doi:10.3109/02813432.2015.1067512. 26375323 PMC4750720

[31] Nordic Federation of General Practice (NFGP). Core values and principles of nordic general practice/family medicine. Scand J Prim Health Care. 2020;38(4):367–368. doi:10.1080/02813432.2020.1842674. 33284030 PMC7782180

[32] National Institute for Health and Care Excellence. Referral guidelines for suspected cancer (clinical guideline 27). NICE, 2005.Available at www.nice.org.uk/guidance/cg27.

[33] Ingeman ML, Christensen MB, Bro F, et al. The danish cancer pathway for patients with serious non-specific symptoms and signs of cancer - a cross-sectional study of patient characteristics and cancer probability. BMC Cancer. 2015;15(1):421. doi:10.1186/s12885-015-1424-5. 25990247 PMC4445271

[34] Sutton RT, Pincock D, Baumgart DC, et al. An overview of clinical decision support systems: benefits, risks, and strategies for success. NPJ Digit Med. 2020;3(1):17. doi:10.1038/s41746-020-0221-y. 32047862 PMC7005290

[35] Jones OT, Calanzani N, Saji S, et al. Artificial intelligence techniques that may be applied to primary care data to facilitate earlier diagnosis of cancer: systematic review. J Med Internet Res. 2021;23(3):e23483. doi:10.2196/23483. 33656443 PMC7970165

[36] Buntinx F, Mant D, Van den Bruel A, et al. Dealing with low-incidence serious diseases in general practice. Br J Gen Pract. 2011;61(582):43–46. doi:10.3399/bjgp11X548974. 21401991 PMC3020049

